# Performance enhancement of a brain-computer interface using high-density multi-distance NIRS

**DOI:** 10.1038/s41598-017-16639-0

**Published:** 2017-11-29

**Authors:** Jaeyoung Shin, Jinuk Kwon, Jongkwan Choi, Chang-Hwan Im

**Affiliations:** 1 Department of Biomedical Engineering, Hanyang University, Seoul, Korea; 2 OBELAB, Seoul, Korea

## Abstract

This study investigated the effectiveness of using a high-density multi-distance source-detector (SD) separations in near-infrared spectroscopy (NIRS), for enhancing the performance of a functional NIRS (fNIRS)-based brain-computer interface (BCI). The NIRS system that was used for the experiment was capable of measuring signals from four SD separations: 15, 21.2, 30, and 33.5 mm, and this allowed the measurement of hemodynamic response alterations at various depths. Fifteen participants were asked to perform mental arithmetic and word chain tasks, to induce task-related hemodynamic response variations, or they were asked to stay relaxed to acquire a baseline signal. To evaluate the degree of BCI performance enhancement by high-density channel configuration, the classification accuracy obtained using a typical low-density lattice SD arrangement, was compared to that obtained using the high-density SD arrangement, while maintaining the SD separation at 30 mm. The analysis results demonstrated that the use of a high-density channel configuration did not result in a noticeable enhancement of classification accuracy. However, the combination of hemodynamic variations, measured by two multi-distance SD separations, resulted in the significant enhancement of overall classification accuracy. The results of this study indicated that the use of high-density multi-distance SD separations can likely provide a new method for enhancing the performance of an fNIRS-BCI.

## Introduction

Near-infrared spectroscopy (NIRS) is becoming a highly popular modality for implementing brain-computer interfaces (BCIs), due to its safety, portability, and high reliability^1^. From the early developmental stage of functional NIRS (fNIRS)-BCI, motor imagery has been most widely adopted as a target mental task^2–4^. However, as the intensity of the light incident on the brain is severely influenced by hair covering the motor-related areas, time-consuming preparatory work is required to prevent the attenuation of light intensity. To avoid this inconvenience, BCI paradigms using the hemodynamic variations of the prefrontal cortex (PFC), which is not covered with hair, have also been actively studied. Among the various mental tasks adopted for these fNIRS-BCIs, the mental arithmetic (MA) task has been widely used because users find it intuitive, and because it demonstrates high reproducibility in terms of induced PFC activations^5–12^.

A common physical constraint, in the majority of fNIRS-BCI studies, is light source-detector (SD) separation. This constraint occurs because the SD separation determines the depth of light penetration through brain tissues. If the SD separation is shortened, the light does not penetrate deeply into the cerebral cortex, whereas, if it is substantially lengthened, the intensity of the light is severely attenuated. Thus, it may not be feasible to obtain useful information on brain activations. For the best classification results, optimal SD separation can be determined by observing all possible depth-dependent hemodynamic variations via a brute-force approach. However, this approach would require heavy computation, as well as an NIRS system with dense optode arrangements, which would make the application of fNIRS-BCI difficult to implement in practical scenarios. Alternatively, as Yamamoto *et al*.^13^ reported, 30 mm may be the most suitable SD separation distance for measuring cortical hemodynamic variations. An SD separation distance of 30 mm has been regarded as standard in the majority of fNIRS-BCI studies. Recently, there has been no meaningful attempt to measure cortical depth-dependent, task-related, hemodynamic variations, particularly in the field of fNIRS-BCI. Furthermore, Gagnon *et al*.^14^, and Brigadoi *et al*.^15^, have recently used substantially short SD separation distances (less than 10 mm) in order to remove superficial physiological noises; however, these studies have not measured task-related hemodynamic variations, and thus they are not related to BCI.

Owing to the limitation of scalability (i.e., constraint in SD separation), the implementation of multi-distance SD separations, within a limited area, has been generally considered as challenging. Recently, an NIRS device consisting of high-density multi-distance SD separations was introduced^16,17^. By using the high-density SD arrangement, cortical depth-dependent hemodynamic variations could be measured with a higher spatial resolution, which enabled the collection of a larger amount of information on cortical activity.

Hemodynamic variations, measured at various cortical depths, may contain not only correlated information but also uncorrelated information. As shown in Fazli *et al*.’s study^18^, the various features containing uncorrelated pieces of information could enhance overall classification accuracy, because the features could work complementarily to each other. To take advantage of this benefit, a hybrid BCI was introduced, and used different brain-imaging modalities simultaneously, to enhance classification performance. Similar to this, different features extracted from various depth-dependent hemodynamic variations, would work complementarily to each other, and could thus lead to a synergetic effect on the enhancement of classification accuracy.

Hence, we anticipated that the spatial sampling increment would likely enhance he overall performance of fNIRS-BCI. However, there has been no study on whether the new type of NIRS device, with high-density multi-distance SD separations, could result in enhanced BCI performance, and particularly in terms of classification accuracy. To the best of our knowledge, this is the first study to investigate the effectiveness of using high-density multi-distance SD separations to record hemodynamic responses in the PFC, for enhancing the performance of an fNIRS-BCI.

## Materials and Methods

### Participants

Fifteen healthy volunteers (six males and nine females, with a mean age of 22.5 ± 2.1 years) participated in this study. None of them reported a previous history of neurological, psychiatric, or other severe diseases, which could likely influence the experimental results. The experimental procedure was thoroughly explained to each volunteer and each participant signed a written consent prior to their participation in the experiment. After the experiment, the volunteers received monetary reimbursement for their participation. The study was reviewed and approved by the Institutional Review Board (IRB) committee of Hanyang University, and was conducted according to the Declaration of Helsinki.

### High-density multi-distance NIRS

**Figure 1:**
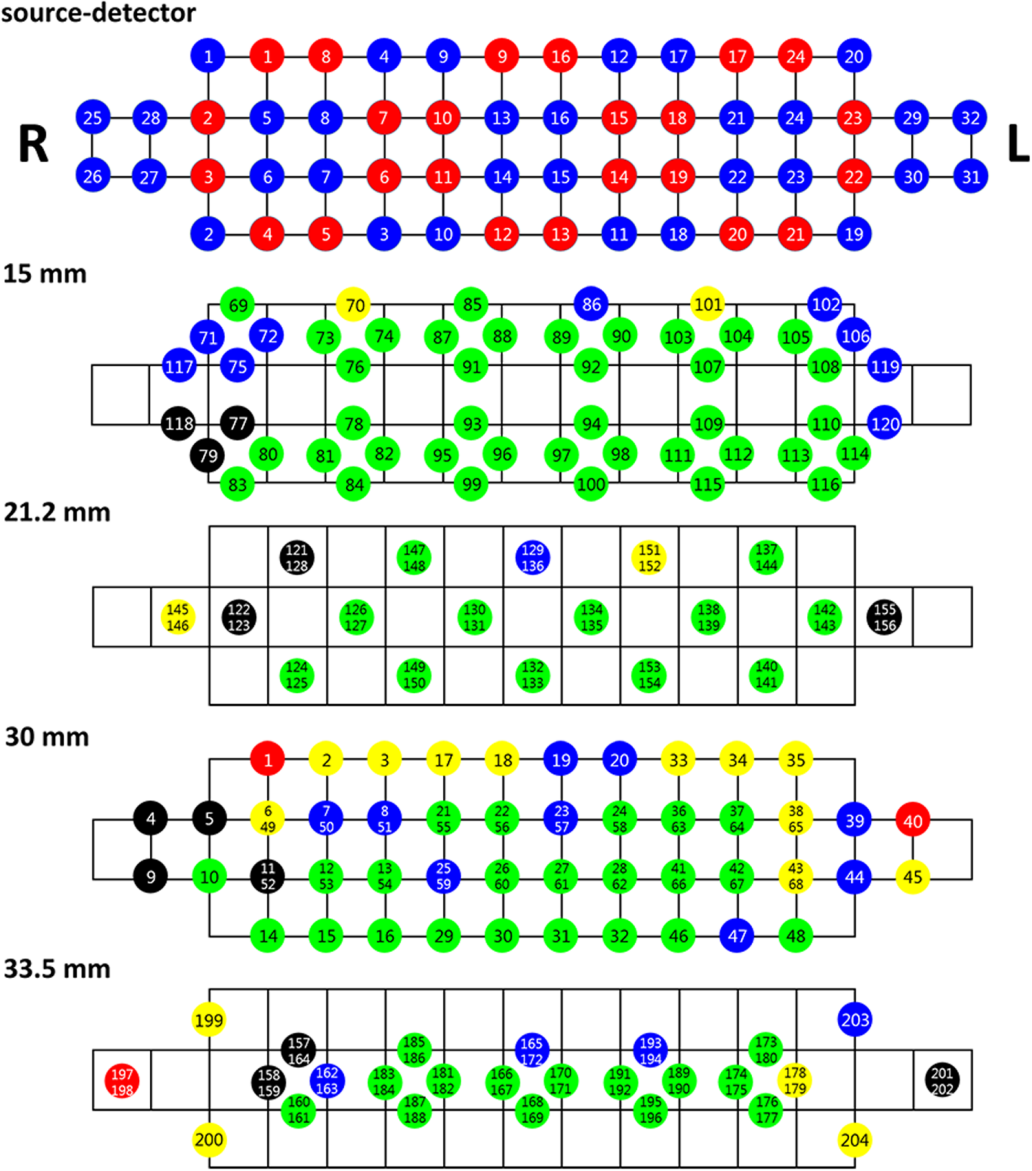
Arrangement of sources and detectors, and location of NIRS channels for 15, 21.2, 30, and 33.5 mm SD separations. The numbers in red and blue circles are source and detector numbers, respectively (top). For numbered NIRS channels, green, blue, yellow, red, and black colours, indicate 0, 0–10, 10–30, 30–50, and higher than 50% rejection ratios, respectively. The size of the grid is 15 × 15 mm.

Hemodynamic variations in the PFC were recorded using a high-density NIRS device (NIRSIT; OBELAB, Seoul, Korea), which was composed of 24 sources (laser diodes) and 32 detectors, at a sampling rate of 8.138 Hz. Adjacent SD pairs could configure 204 channels. Figure 1 illustrates the SD arrangement (top figure) and channel locations, with respect to various SD separations. As depicted in the figure, various pairings of sources and detectors could make four SD separations. The nearest SD pair (e.g., S1-D1 and S2-D2) configured a 15 mm SD separation, and the diagonally positioned SD pair configured a 21.2 mm SD separation (e.g., S1-D8 and S8-D5). The lattice-arranged SD pair (e.g., S1-D6 and S2-D8) corresponded to a 30 mm SD separation. Finally, the SD pair, whose distance was the same as that of S2-D7 and S8-D6, formed a 33.5 mm SD separation. When the SD separations were 15, 21.2, 30, and 33.5 mm, the numbers of channels were 52, 36 (18 channels +18 overlapped channels), 68 (48 channels +20 overlapped channels), and 48 (26 channels +22 overlapped channels), respectively. One of the overlapped channels was selected and the rest of overlapped channels were disregarded.

### Experimental conditions

**Figure 2:**
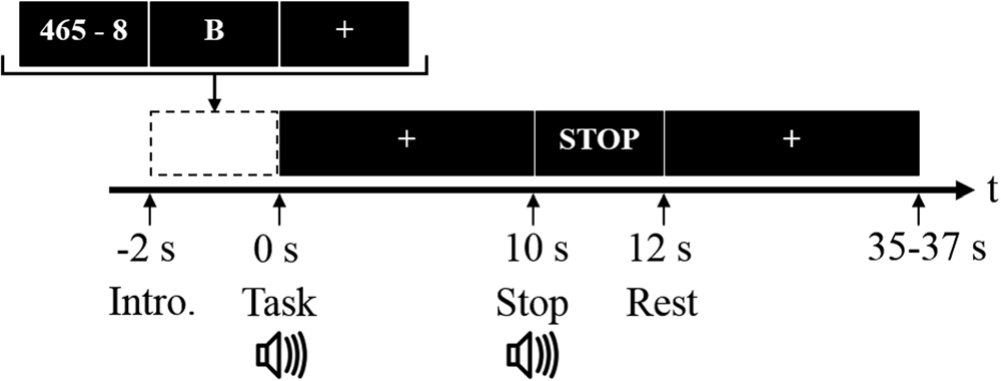
Schematic diagram of the experimental paradigm. At the instruction period (−2 to 0 s), a calculation problem (random three-digit number minus a one-digit number between six and nine; e.g., 465 - 8), a random single letter (e.g., B), or a fixation cross, was randomly presented for each MA, WC, or BL trial, respectively. Subsequent to the instruction period, the participants performed the assigned task during the task period (0 to 10 s), while observing the fixation cross. The task period ended with a ‘STOP’ sign being displayed on the screen, and was followed by a resting period, which varied randomly from 25 to 27 s. At the beginning and end of the task period, a short beep sound was played.

Each participant sat on a comfortable chair, located 70 cm away from a 24″ LED monitor. The experiment was conducted over three sessions involving three mental tasks; namely, an MA task, word chain (WC) task, and baseline (BL) task. In the beginning of each session, the participants relaxed while observing a fixation cross, which was displayed at the centre of the monitor for 2 min. Figure 2 illustrates a schematic diagram of the experimental paradigm. A single session consisted of 30 randomized trials (10 repetitions for each task), each of which started with a 2 s long visual introduction of the task. During the instruction period, a calculation problem (random three-digit number minus a one-digit number), a random single letter, or the fixation cross, were presented randomly, and corresponding to each MA, WC, or BL trial, respectively. After the instruction period, the participants performed the designated mental task for 10 s, while observing the fixation cross. For the MA task, the participants were instructed to continuously subtract a one-digit number (between six and nine), as fast as they could, from the results of the former calculation (e.g., 789–7 = 782, 782–7 = 775, 775–7 = 768). For the WC task, the participants were instructed to continuously suggest a word starting with the last character of the former word (e.g., in English, B: Boy – year – rabbit – tree) as fast as they could. In each trial the participants were instructed to not repeat previously used words. The WC task was performed in the participants’ native language (Korean). For the BL task, the participants relaxed without any distracting thoughts.

The task period ended with a ‘STOP’ sign displayed on the screen, and followed by a resting period, which varied randomly from 25 to 27 s, to avoid potential adaptation to the task routine. At the beginning and end of the task period, a short pure-tone beep sound was played through a loudspeaker.

### Pre-processing

Data analyses were performed using MATLAB R2013b (MathWorks, Natick, MA, USA). The raw light-intensity data were low-pass filtered at 0.5 Hz, with a sixth order zero-phase Butterworth filter. Owing to the signal interference due to hair around the forehead, and the incomplete contact of a few optodes, the signal quality of certain channels was not adequate for use in further analysis. Therefore, a few channels with inadequate signal quality were rejected if they did not meet the following criteria:

1 $${\rm{coefficient}}\,{\rm{of}}\,{\rm{variance}}\,({\rm{CV}})=100\times \frac{\sigma }{\mu } < 40$$

2 $${\rm{light}}\,{\rm{intensity}} > 10$$

where *μ* and *σ* are the mean and standard deviation, respectively, of the signal recorded during a single session. If a certain channel did not meet one or two of the above criteria, in any of the three sessions, the channel was not used in subsequent analyses. In Figure 1, which illustrates the channel locations, the colour within each circle represents the rejection ratio for the entire set of participants (i.e., number of rejections/number of participants). Green, blue, yellow, red, and black, indicate rejection ratios of 0, 0–10, 10–30, 30–50, and over 50%, respectively. The variations in the concentrations of deoxy- and oxy-haemoglobin (ΔHbR and ΔHbO) were calculated using the modified Beer–Lambert law^19^. Then, both chromophore data were band-pass filtered at 0.01–0.09 Hz, with a sixth order zero-phase Butterworth filter, in order to eliminate systemic noise^20^.

### Classification

For the classification of the various mental tasks, a feature vector was constructed using the average hemodynamic variations (both ΔHbR and ΔHbO) of the remaining channels over three time windows; namely, 0–5, 5–10, and 10–15 s, after the rejection of ineffective channels. A 10 × 5-fold cross-validation was carried out by using shrinkage linear discriminant analysis (sLDA). sLDA is known to enhance the estimation accuracy of covariance matrices, if the number of measured samples is small, in comparison to the dimension of the feature vector^21–23^. First, it was investigated whether the use of a high-density channel configuration could enhance classification accuracy, in comparison to the classification accuracy achieved by using the typical low-density channel configuration, while maintaining the SD separation at 30 mm. The fourth image of Figure 1 (SD separation = 30 mm) illustrates the high-density channel configuration, when all the SD pairs, with an SD separation of 30 mm, were used. On the other hand, for the low-density typical LA configuration, a few of the sources and detectors were selectively used (see supplementary Figure 1 for the selected source/detectors and the resultant channel configuration). Note that the SD separation was set to 30 mm, in both the high-density and low-density NIRS channel configurations, and that the only variation was in channel density.

**Figure 3:**
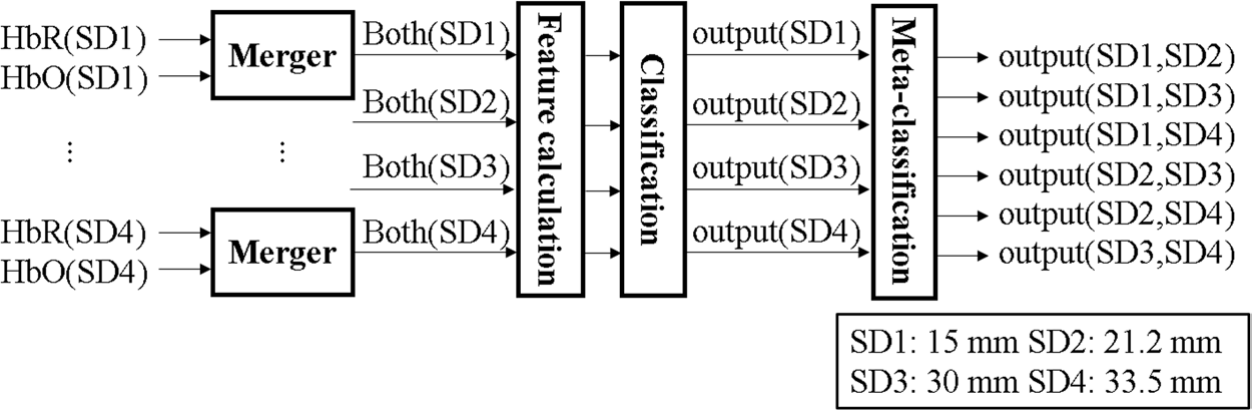
Data processing flow for classification. A feature vector was constructed by concatenating HbR and HbO, for the channels that remained after channel rejection. The classifier outputs, for each SD separation, were concatenated in pairs, to construct a feature vector for meta-classification.

Subsequently, the classification accuracy was calculated when the two channel configurations, formed by various SD separations, were simultaneously used for the classification of mental tasks. A meta-classification method^24^, whose schematic diagram is depicted in Figure 3, was used to evaluate the classification accuracies for six paired combinations of multi-distance SD separations (i.e., {15 and 21.2}, {15 and 30}, {15 and 33.5}, {21.2 and 3}, {21.2 and 33.5}, and {30 and 33.5}). In the meta-classification method, the classifier outputs for each SD separation, were combined in a pairwise manner, to construct a new feature vector, which was then utilized by the meta-classifier to calculate the classification accuracy for the combined SD separation. Here, we briefly introduce the meta-classification method:

1) Set feature vectors, **x**_**1**_ and **x**_**2**_ for the first and second SD separations, respectively.

2) Classifier output, **y**_**1**_ for the first SD separation: ${{\bf{y}}}_{1}={{\bf{w}}}_{1}^{{\bf{T}}}{{\bf{x}}}_{1}+{{\bf{b}}}_{1}$

3) Classifier output, **y**_**2**_ for the second SD separation: ${{\bf{y}}}_{2}={{\bf{w}}}_{2}^{{\bf{T}}}{{\bf{x}}}_{2}+{{\bf{b}}}_{2}$

4) Meta-classifier input: **x**_**m**_ = [**y**_**1**_, **y**_**2**_]

5) Meta-classifier output: ${{\bf{y}}}_{{\bf{m}}}={{\bf{w}}}_{{\bf{m}}}^{{\bf{T}}}{{\bf{x}}}_{{\bf{m}}}+{{\bf{b}}}_{{\bf{m}}}$

$${\bf{c}}{\bf{l}}{\bf{a}}{\bf{s}}{\bf{s}}=\{\begin{array}{c}0,\,\,{{\bf{y}}}_{{\bf{m}}} < 0\\ 1,\,{{\bf{y}}}_{{\bf{m}}} > 0\end{array}$$

A detailed description of the meta-classification method is available in the literature^24^.

### Data availability

All relevant data are available at the following Figshare DOI: 10.6084/m9.figshare.5606029.

## Results

### Hemodynamic variations

**Figure 4:**
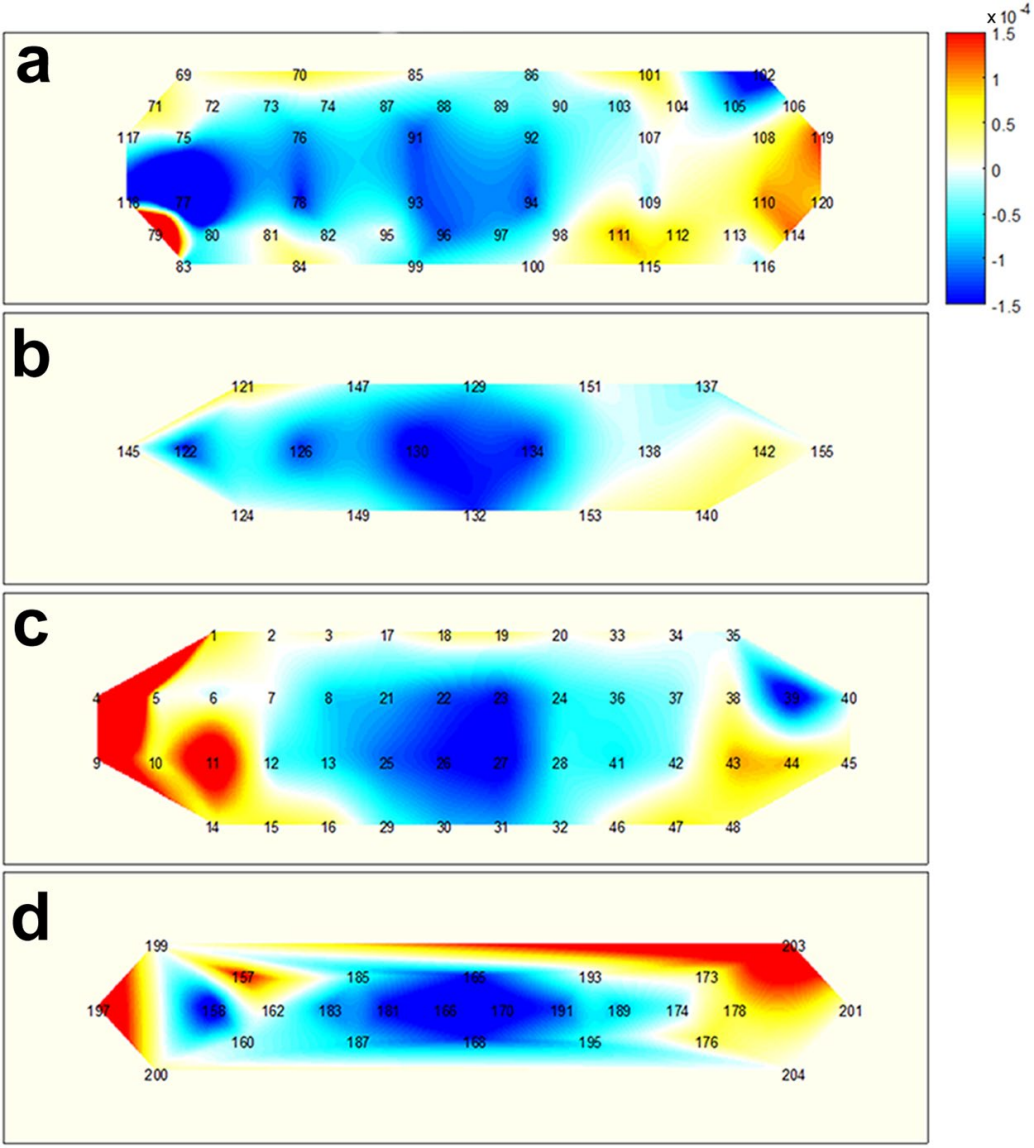
Hemodynamic variations (ΔHbO) induced by MA and measured by using four SD separations 15 s after task onset: (**a**) 15 mm, (**b**) 21.2 mm, (**c**) 30 mm, and (**d**) 33.5 mm. The numbers in each subfigure denote the locations of NIRS channels. The vertical colour bar indicates the range in mmol/L.

**Figure 5:**
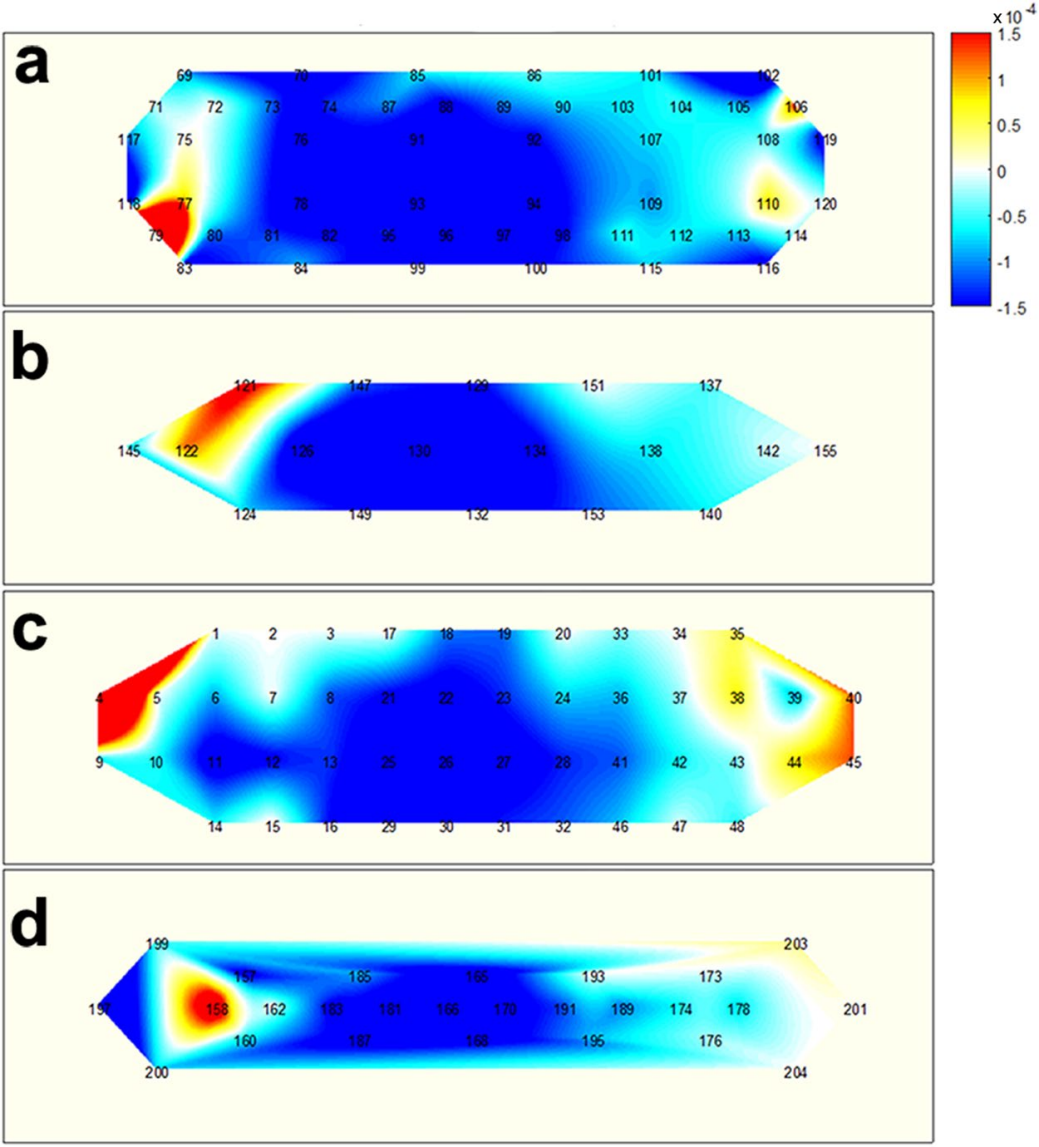
Hemodynamic variations (ΔHbO) induced by WC and measured by using four SD separations 15 s after task onset: (**a**) 15 mm, (**b**) 21.2 mm, (**c**) 30 mm, and (**d**) 33.5 mm. The numbers in each subfigure denote the locations of NIRS channels. The vertical colour bar indicates the range in mmol/L.

**Figure 6:**
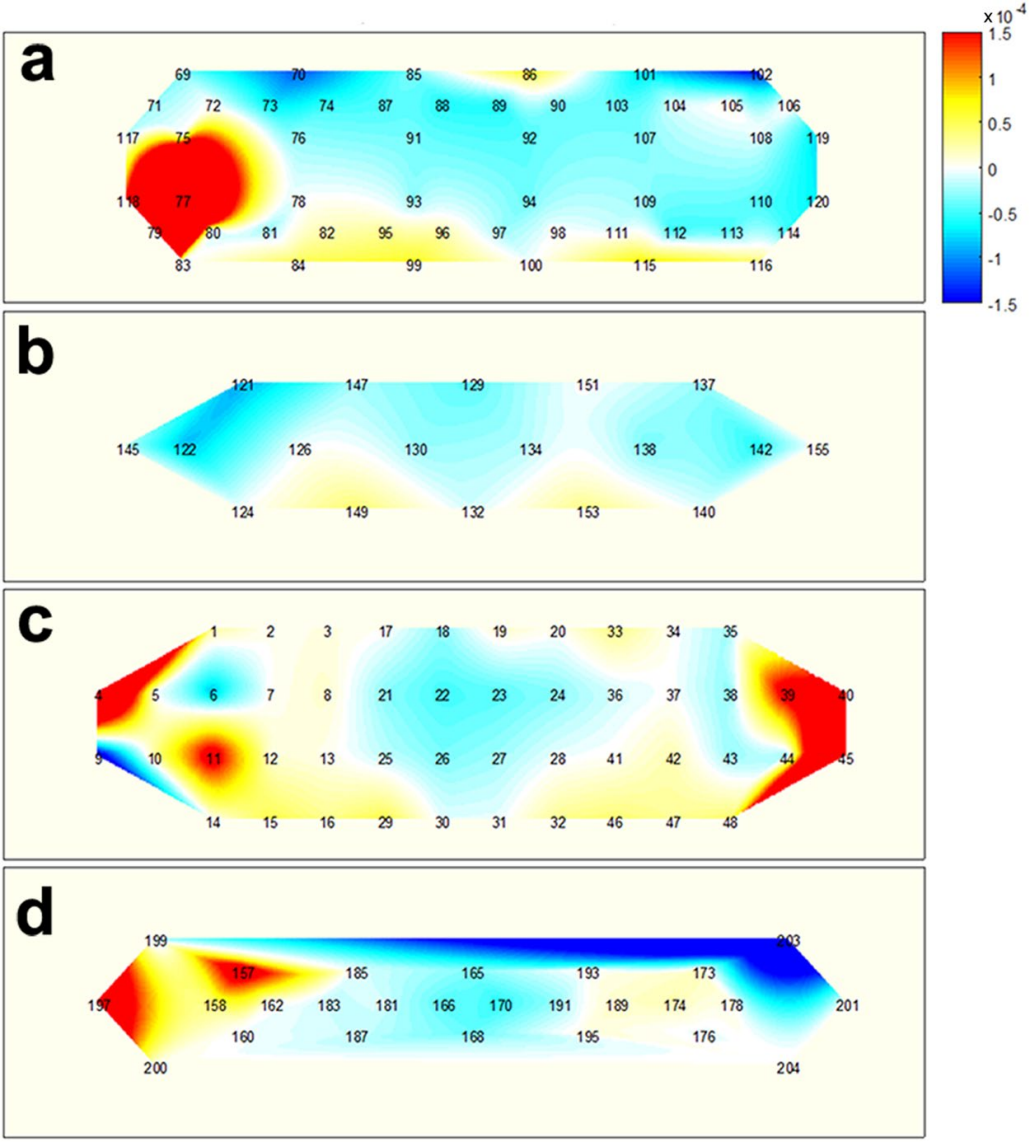
Hemodynamic variations (ΔHbO) induced by BL and measured by using four SD separations 15 s after task onset: (**a**) 15 mm, (**b**) 21.2 mm, (**c**) 30 mm, and (**d**) 33.5 mm. The numbers in each subfigure denote the locations of NIRS channels. The vertical colour bar indicates the range in mmol/L.

Figures 4–6 illustrate the average task-related (MA, WC, and BL, respectively) hemodynamic response variations (ΔHbO as a representative), which were grand averaged over all the participants at 15 s from the task onset time. Note that channels, which were not considered in the classification, due to their low signal quality, were used to calculate the grand average. The numbers shown in each subfigure denote the locations of the NIRS channels, as depicted in Figure 1. During the MA Task (Figure 4), a decrease in ΔHbO was observed in the centrally located channels, regardless of the SD pair distance. This was in accordance to previously reported observations^5^. The prominent hemodynamic variations observed in channel 11, in Figure 4c, were not likely to represent meaningful brain activation, since the signal quality of the channel was bad due to noise contamination and the loss in contact of optodes, (see the black colour of the channel in Figure 1). During the WC task (Figure 5), a relatively wider and stronger decrease in the concentration of oxy-haemoglobin was observed in centrally located channels, when compared to the distributions depicted in Figure 4. A few singular points (e.g., channel 158 in Figure 5d) are also likely to have originated from signal distortion. A noticeable hemodynamic variation was not observed in Figure 6, because the participants relaxed and did not perform any of the mental tasks (neither MA nor WC).

### Low-density NIRS vs. high-density NIRS

**Figure 7:**
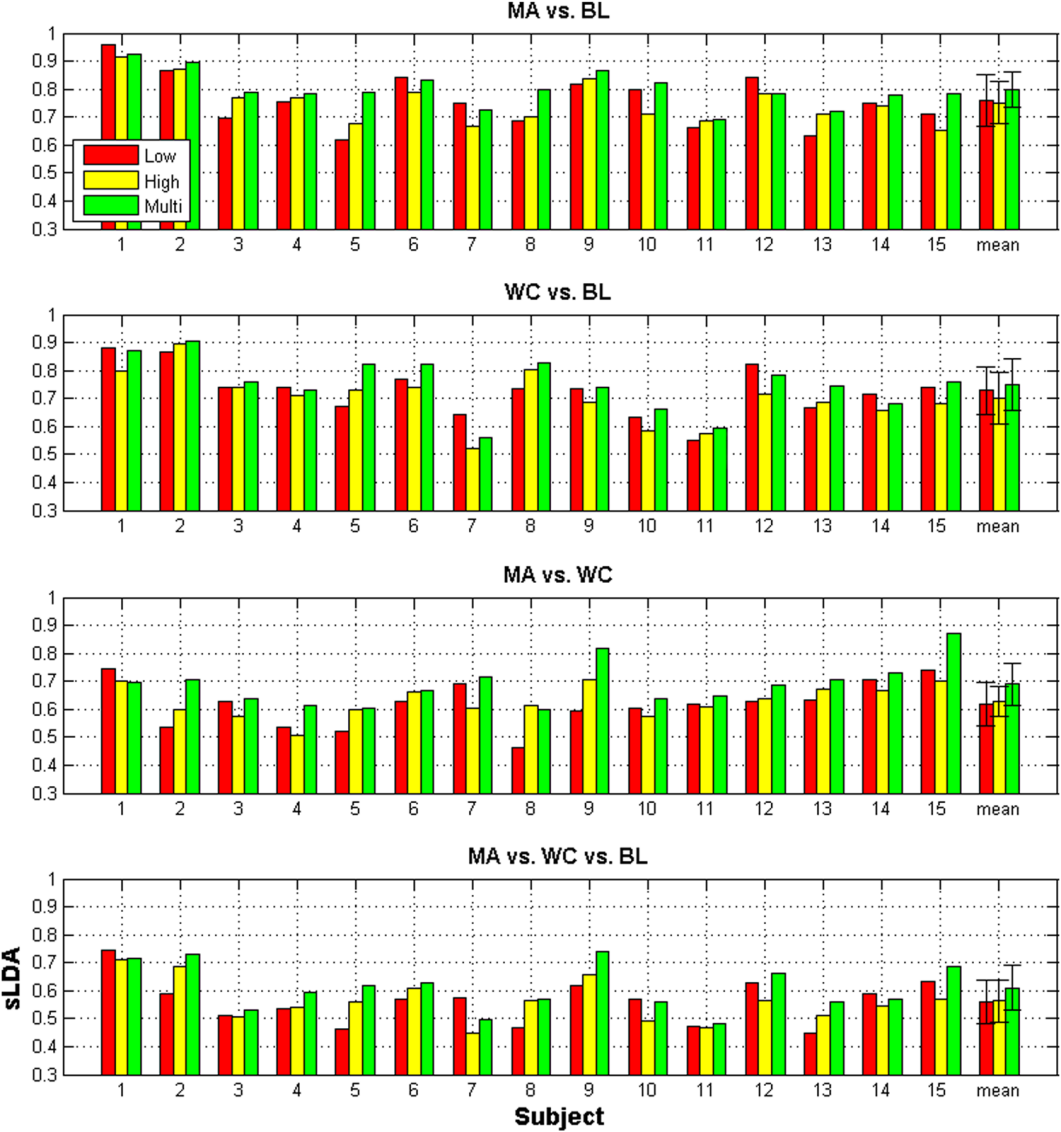
Comparison of binary and ternary classification accuracies for the lattice arrangement of the 30 mm SD separation (denoted by Low), high-density 30 mm SD separation (denoted by High), and combined multi-distance SD separations (denoted by Multi). The highest classification accuracy occurs among the classification accuracies of all possible multi-distance SD separations. The error bar indicates the standard deviation.

First, it was investigated whether the use of a high-density channel configuration could enhance the classification accuracy of an fNIRS-BCI, from the accuracy obtained by using a typical low-density channel configuration. Figure 7 shows the comparison of binary and ternary classification accuracy, with respect to channel density, where ‘Low’ and ‘High’ indicate low-density and high-density channel configurations, respectively. The MA vs. BL tasks, and the WC vs. BL tasks, could be classified with an accuracy higher than 70%, on average^25,26^, which is fairly reasonable, while the MA vs. WC tasks were classified with a classification accuracy of approximately 60%. In the case of ternary classification, a classification accuracy of less than 60% was achieved. Two out of fifteen participants demonstrated a classification accuracy higher than 70%. For both MA vs. BL and WC vs. BL classifications, typical LA configuration displayed a marginally higher classification accuracy, in comparison to high-density channel configuration, while the high-density channel configuration displayed a marginally higher classification accuracy in MA vs. WC and ternary classifications. Nonetheless, none of the results were statistically significant (Wilcoxon rank test with Bonferroni-correction: p = 0.679, for MA vs. BL, and p = 0.109 for WC vs. BL).

### Single SD vs. multi-distance SD

All the possible pairs of SD separations were considered in the construction of various feature vectors. Then, the pair which yielded the highest classification accuracy was selected. The resultant classification accuracy, obtained by combining multi-distance SD separations (denoted by ‘Multi’), was also compared to those from low-density and high-density channel configurations shown in Figure 7. By comparing the single high-density SD separation (High) to the multi-distance SD separations (Multi), both binary (average of MA vs. BL, WC vs. BL, and MA vs. WC) and the ternary classification accuracies were enhanced by 5.2% and 4.7%, respectively. It is noteworthy that both binary and ternary classification accuracies were significantly enhanced by using multi-distance SD separations, in comparison to using single SD separation (Wilcoxon rank test with Bonferroni-correction: corrected p <0.05).

**Figure 8:**
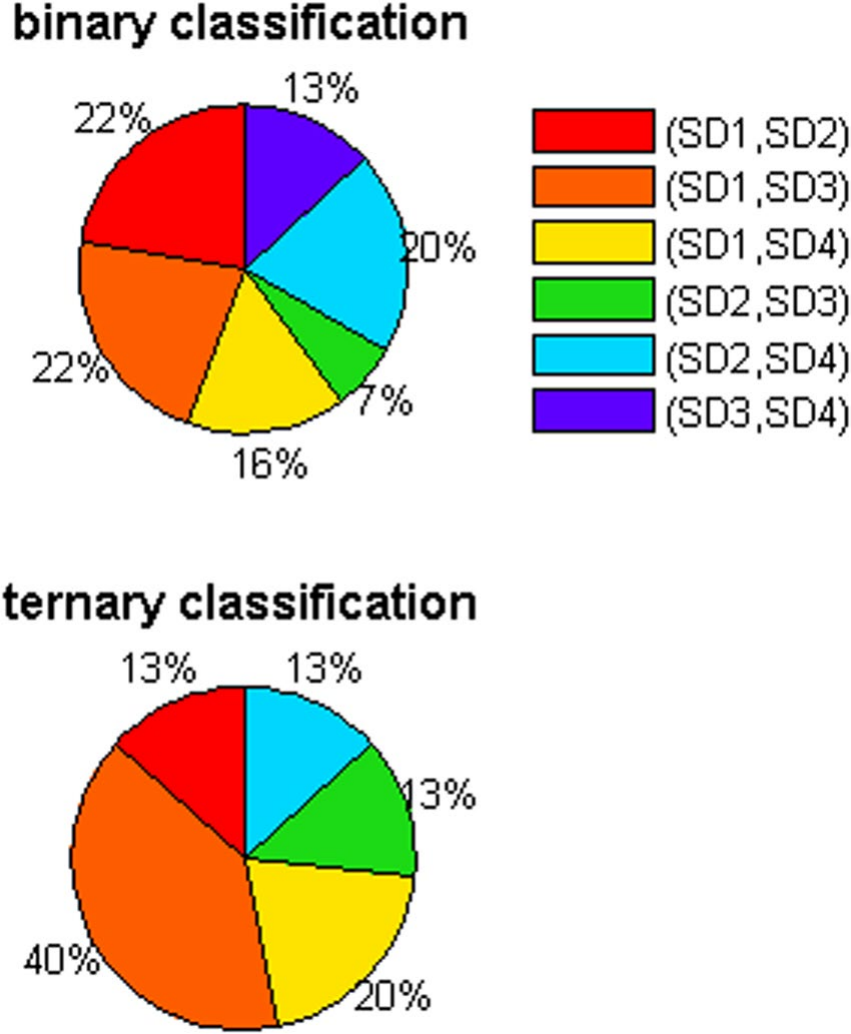
Selection frequency of multi-distance SD separations representing the highest classification accuracy in Figure 7.

Figure 8 illustrates the frequency with which each pair of multi-distance SD separations was selected as having the highest classification accuracy. The SD separations are denoted by SD1 (15 mm), SD2 (21.2 mm), SD3 (30 mm), and SD4 (33.5 mm). For binary classification, the highest classification accuracy was most frequently derived from two combinations of the SD separations; namely, (SD1, SD2) and (SD1, SD3), while the combination (SD2, SD3) was selected least frequently. Apart from (SD2, SD3), the selection frequency was largely unbiased. For the ternary classification, (SD1, SD3) was selected most frequently, while (SD3, SD4) was selected least frequently. The rest of the cases exhibited a more or less uniform frequency.

## Discussion

In this study, we investigated whether the use of NIRS, with high-density multi-distance SD separations for fNIRS-BCI, could result in enhanced BCI performance. First, the enhancement of BCI performance, by high-density NIRS recording, was evaluated. Contrary to our hypothesis, the straightforward use of higher density recording data did not result in the significant enhancement of BCI performance. On the other hand, BCI performance was significantly enhanced by combining two channel configurations corresponding to different SD separations.

NIRS-related brain-imaging and neuroscience has been actively investigated. Nowadays, BCI has become an emerging application field, which receives a great amount of attention. Up to now, existing approaches toward enhancing overall classification accuracy have been mainly based on the suggestion of advanced algorithms, such as robust and optimal feature extraction methods, and classification strategies. In this study, it was demonstrated that classification accuracy could be enhanced by incorporating new information from depth-dependent hemodynamic variations, and in particular by those measured using short SD separations (shorter than the typically used SD separation distance of 30 mm). In previous studies, similar short SD separations were used only to remove global physiological noises; however, the results of this study demonstrated that the classification accuracy could be enhanced by using short SDs of 15 and 21.2 mm, for example.

The separation distance of 30 mm has been considered as the optimal SD separation distance for measuring cerebral hemodynamic variations. However, according to Brigadoi *et al*.^15^ and Strangman *et al*.^27^, it is feasible to measure hemodynamic variations in the outermost superficial layer of the cerebral cortex, with a short SD separation distance of 15 or 21.2 mm, for example. Since the information from the superficial cortical layer is likely to vary from that obtained with the typical 30 mm SD separation, the use of multiple SD separations is likely to be effective for enhancing the performance of fNIRS-BCI. Although it has been reported that task-related hemodynamic variations can also be measured by using a short SD separation (e.g., Funane *et al*.^28^), the hemodynamic response variations measured using short SD separations are likely to mainly reflect secondary systemic variations, such as cerebral blood flow and volume variations, rather than the direct task-related hemodynamic variations induced by neurovascular coupling. It has been demonstrated by Zhang *et al*.^29^, and Scholkmann *et al*.^20^, that systemic variations can occur secondarily, as a result of performing certain mental tasks.

In our study, we investigated whether pairwise combinations of four SD separations could lead to the enhancement of fNIRS-BCI performance. We also investigated whether the use of all four sorts of multi-distance SD separations could yield improved classification accuracy; however, we could not find any notable improvements compared with the pairwise combination of multi-distance SD separations. Figure S2 in the supplementary information shows the comparison of classification accuracies for the two cases, namely the 30 mm SD separation and the combination of all four multi-distance SD separations. For all four cases (three binary classifications and a ternary classification), although the classification accuracy was slightly increased by the combined use of the multi-distance SD separations, the difference was not statistically significant (Wilcoxson signed rank sum test, p > 0.05, for all cases). This result suggests that the combination of all four multi-distance SD separations is not effective to improve the classification accuracy, but it just increases the computational complexity.

The combined use of multi-distance SD separations is conceptually similar to the hybrid BCI, with respect to new information being incorporated to enhance BCI performance^12,24^. Note that the meta-classification method, used in this study for classification with the combined multi-distance SD separations, was adopted from a hybrid BCI study^24^. To set up a hybrid BCI, separate equipment is generally required for measuring brain- and bio-signals, which results in an increase of preparation time, system size, and experimental complexity. In contrast, the use of multi-distance SD separations requires a single NIRS device.

For the combination of multi-distance SD separation to be utilized for practical real-time fNIRS-BCI, some technical issues need to be considered. First, the optimal SD separation pair showing the best classification accuracy is individually different. In the present offline study, the optimum was obtained from the comparison of classification accuracies for pairwise combined SD separations; however, there is a need to set up more concrete criteria for the selection of an optimum combination for practical applications. Secondly, when calibrating the channels corresponding to all SD separations, the strengthening of light intensity in order to increase the signal-to-noise ratio of the channels being configured by long SD separations (e.g., 30 or 33.5 mm in this study), may cause signal saturation to channels corresponding to short SD separation^30^ (e.g., 15 mm in this study). Therefore, we may not properly obtain the NIRS signals, corresponding to the desired SD separation, and the calibration needs to be performed repeatedly to get the desired signals. Thus, methods to determine the proper light intensity need to be developed to apply the multi-distance SD separations in practical BCI scenarios.

## Conclusion

In this study, the feasibility of enhancing fNIRS-BCI performance by using an NIRS device with high-density multi-distance SD separations was investigated. Experimental studies with 15 participants demonstrated that the use of the high-density channel configuration did not, in itself, result in a noticeable enhancement in the classification accuracy of mental task-based BCI, when compared to the typical channel configuration. However, the combined use of depth-dependent cortical hemodynamic responses, recorded from multi-distance SD separations, contributed significantly to the enhancement of overall classification accuracy. This was the first study that demonstrated that the classification accuracy of fNIRS-BCI might be significantly enhanced by adopting an advanced NIRS hardware system. It is expected that our new findings would contribute to inspiring new ideas in fNIRS-BCI research.

## Electronic supplementary material

Supplementary Information
