# Supplementary material for: Performance enhancement of a brain-computer interface using high-density multi-distance NIRS
Source: Sci Rep. 2017 Nov 29;7:16545. doi: 10.1038/s41598-017-16639-0 (PMC5707382; doi:10.1038/s41598-017-16639-0)
Supplement: Supplementary file 1 — Supplementary Information [file 41598_2017_16639_MOESM1_ESM.pdf]

Performance Enhancement of a Brain-Computer Interface using High-Density Multi-Distance NIRS

Jaeyoung Shin[1], Jinuk Kwon[1], Jongkwan Choi[2] and Chang-Hwan Im[1*]
[1]Department of Biomedical Engineering, Hanyang University, Seoul, Korea
[2]OBELAB, Seoul, Korea
[*]correspondence: ich@hanyang.ac.kr

Supplementary information

Figure S1 illustrates the low-density typical LA and locations of channels. All sources and detectors were used to implement the high-density SD separation (see Figure 1) while sources and detectors were used selectively to implement the low-density typical LA.

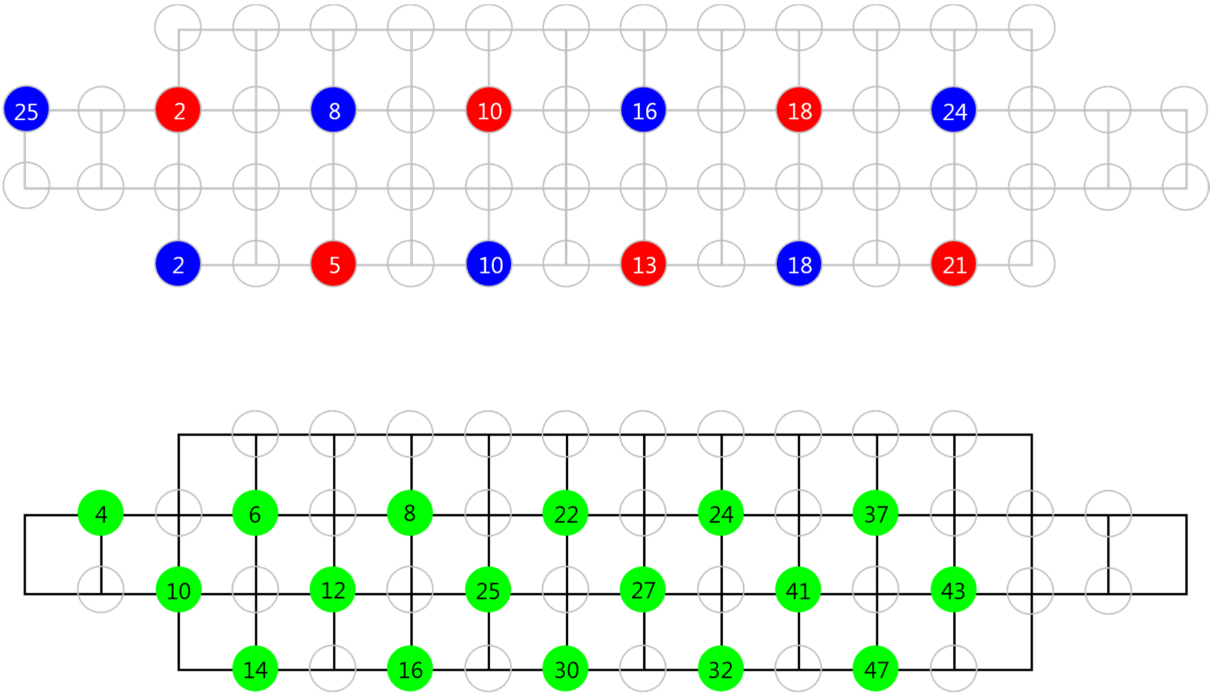

Figure S1. Low-density typical lattice SD arrangement (top) and corresponding NIRS channels (bottom). Red and blue circles are sources and detectors, respectively.

Figure S2 illustrates the comparison between classification accuracies obtained using 30 mm SD separation and the combination of all four SD separations.

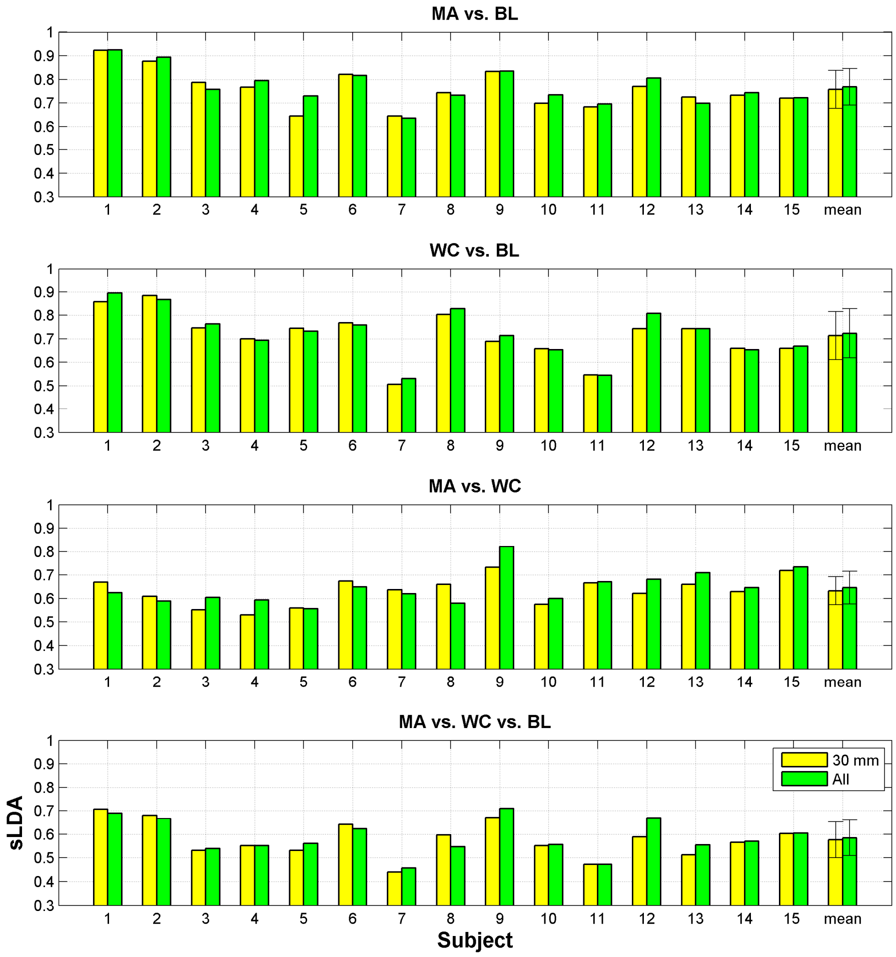

Figure S2. Comparison of classification accuracies for 30 mm SD separation and combination of all four multi-distance SD separations. An errorbar indicates the standard deviation.
